# A review on the research progress of nano-preparations of traditional Chinese medicine for cardiovascular diseases

**DOI:** 10.1097/MD.0000000000045911

**Published:** 2025-11-14

**Authors:** Yuhang Cheng, Miao Yu, Chenwei Dang, Huijun Chen

**Affiliations:** a Heilongjiang University of Traditional Chinese Medicine, Harbin, Heilongjiang, China; b Second Affiliated Hospital of Heilongjiang University of Traditional Chinese Medicine, Harbin, Heilongjiang, China.

**Keywords:** cardiovascular diseases, Chinese medicine based-nanomaterials, coronary, nano science, nanotechnology

## Abstract

Nanomedicine, an emerging therapeutic approach, has shown significant promise for the treatment of coronary heart disease (CHD). Despite advancements in conventional therapies, CHD remains a major cardiovascular health issue, necessitating new and more effective treatment options. This review aimed to comprehensively examine the progress and potential of nanomedicine, particularly traditional Chinese medicine (TCM) nanoparticles, for the treatment of CHD. Background of CHD, including its epidemiological characteristics and the limitations of current therapeutic strategies. Preparation methods and characteristics of TCM nanoparticles: a detailed discussion of the various preparation methods for TCM nanoparticles, including physical, chemical, and biological approaches. This review also covers the key characteristics of these nanoparticles, such as particle size, surface properties, and drug release kinetics. Applications of TCM nanoparticles in treating CHD, a review of the mechanisms by which TCM nanoparticles regulate pathophysiological processes in CHD, enhanced pharmacological effects, and the results of relevant clinical trials. Challenges and prospects in clinical applications: a discussion of the current challenges faced by nanomedicine in clinical settings, including issues related to drug safety, large-scale production, and standardization. The future prospects of nanomedicine in CHD treatment, such as personalized and multimodal therapies, were also explored. This review highlights the potential of nanomedicine, particularly TCM nanoparticles, to provide more effective and personalized treatment options for patients with CHD. This study aims to offer insights and references that can further promote the application of nanomedicine in clinical practice.

## 1. Introduction

Coronary artery disease (CAD) is triggered by coronary atherosclerosis, which leads to narrowed and clogged arteries, resulting in insufficient blood supply, which in turn damages heart tissue.^[1]^ As a significant component of cardiovascular disease worldwide, it continues to pose a major threat to public health.^[2]^ According to WHO data, cardiovascular disease is the major cause of death worldwide, with coronary heart disease (CHD) accounting for a large proportion of the total.^[3]^ Millions of deaths from CHD occur worldwide each year, underscoring the epidemiological severity of CHD.^[4]^ At the regional level, the morbidity and death rates of CHD vary greatly among different countries and regions due to a variety of effects, such as heredity, environment, dietary habits, and lifestyle.^[5,6]^ Major risk factors include smoking, high blood pressure, high cholesterol, diabetes, unhealthy eating habits, and physical inactivity.^[7,8]^ In China, the morbidity and mortality rates of CHD are on the rise.^[9]^ Approximately 330 million people will be affected by cardiovascular disease in 2020, of which 11.39 million will have CAD.^[10]^ The main treatments for CAD include percutaneous coronary intervention (PCI); coronary artery bypass grafting; and prolonged administration of anticoagulants, antiplatelet drugs, and lipid-lowering medications.^[9,11]^ Nevertheless, these treatments may lead to comorbidities, such as angiogenesis, plaque formation, restenosis, and drug-induced systemic toxicity.^[10,12,13]^ Given the high morbidity and mortality rates of CHD and the limitations of traditional treatments, it is particularly important to explore new treatment strategies.

With the advancement of technology, nanotechnology has emerged as a frontier in medical research, showing significant promise for the development of drug delivery systems.^[14,15]^ Nanotechnology enables the precise control and efficient delivery of drugs by manipulating materials at the nanoscale, thereby enhancing therapeutic effects and reducing side effects.^[16,17]^ Traditional Chinese medicine, as a treatment method with a long history, can enhance its bioavailability through the nanonization of its active components, which also improves therapeutic efficiency by enhancing the solubility and stability of the drugs.^[18,19]^ Recent advances have highlighted the critical role of nanobiomaterials in modulating disease progression and therapeutic efficacy. For instance, nanobiomaterial-based strategies have been widely investigated in cardiovascular disease and regenerative medicine, where signaling regulation and gene/cell therapy approaches demonstrated promising translational potential.^[20]^ In parallel, green synthesis of nanoparticles has attracted great attention due to its eco-friendly and biocompatible properties. Plant-derived selenium nanoparticles from *Camellia sinensis* were shown to modulate enzymatic activities,^[21]^ while silver nanoparticles synthesized from natural extracts exhibited antimicrobial, cytotoxic, and radiosensitizing properties in cancer therapy.^[22,23]^ Moreover, polysaccharide-based nanocarriers, such as chitosan formulations incorporating plant extracts, have been developed for biomedical and food conservation applications, underscoring the versatility of natural compound-based nanoplatforms.^[24]^ Collectively, these studies highlight the expanding landscape of nanomaterial-based therapeutics and provide a strong foundation for the design of our current nanosystem.

**Figure 1.**
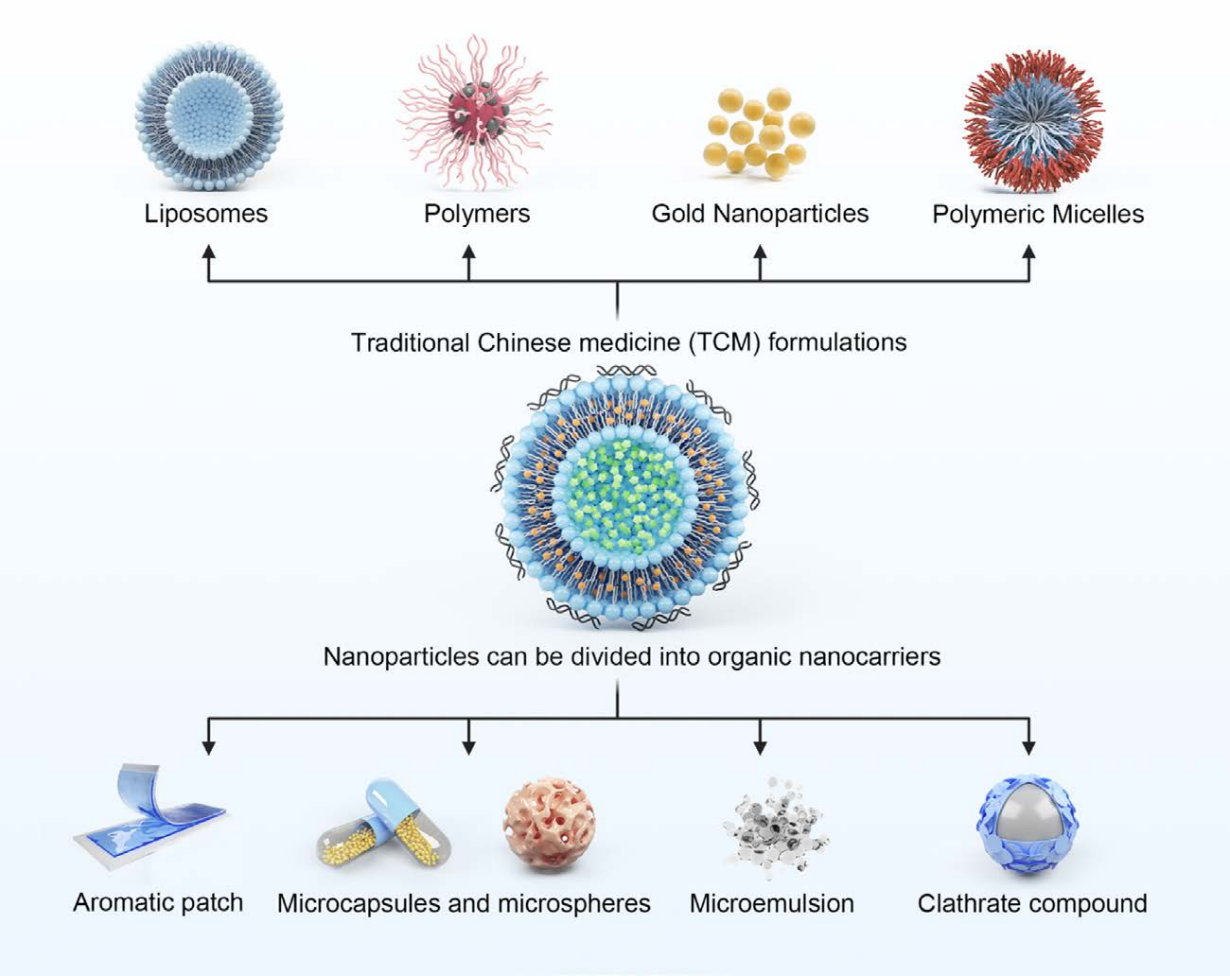
The way in which nanoparticles work on coronary artery disease.

In recent years, traditional Chinese medicine (TCM) nanoparticles have provided new possibilities for the treatment of CHDs owing to their unique advantages.^[25]^ The application of nanomedicine is not only expected to enhance the therapeutic efficacy of drugs, but also to decrease adverse effects through targeted drug delivery, thus playing an important role in treating CHD.^[26,27]^ This review highlights the application of nanoparticles mediated drug delivery systems in combination with TCM for the treatment of CAD. With the benefits of targeted drug delivery, biodegradability, low toxicity, and good biocompatibility, nanoparticles have shown great potential as drug carriers.^[28,29]^ In the medical field, nanotechnology has a variety of uses, ranging from clinical imaging and diagnostics to drug and gene delivery systems and tissue-engineered scaffolds.^[30,31]^ Therefore, reviewing the development of nanomedicine for treating CHD is not only a summary of existing research results but also an exploration of future research directions.^[14,32]^ Through this review, we aimed to evaluate the effectiveness and safety of nanomedicines in treating CHD in a systematic manner and to provide a theoretical basis and practical guidance for further research and clinical applications (Fig. 1).

## 2. Materials and methods

### 2.1. Literature search strategy

**Figure 2.**
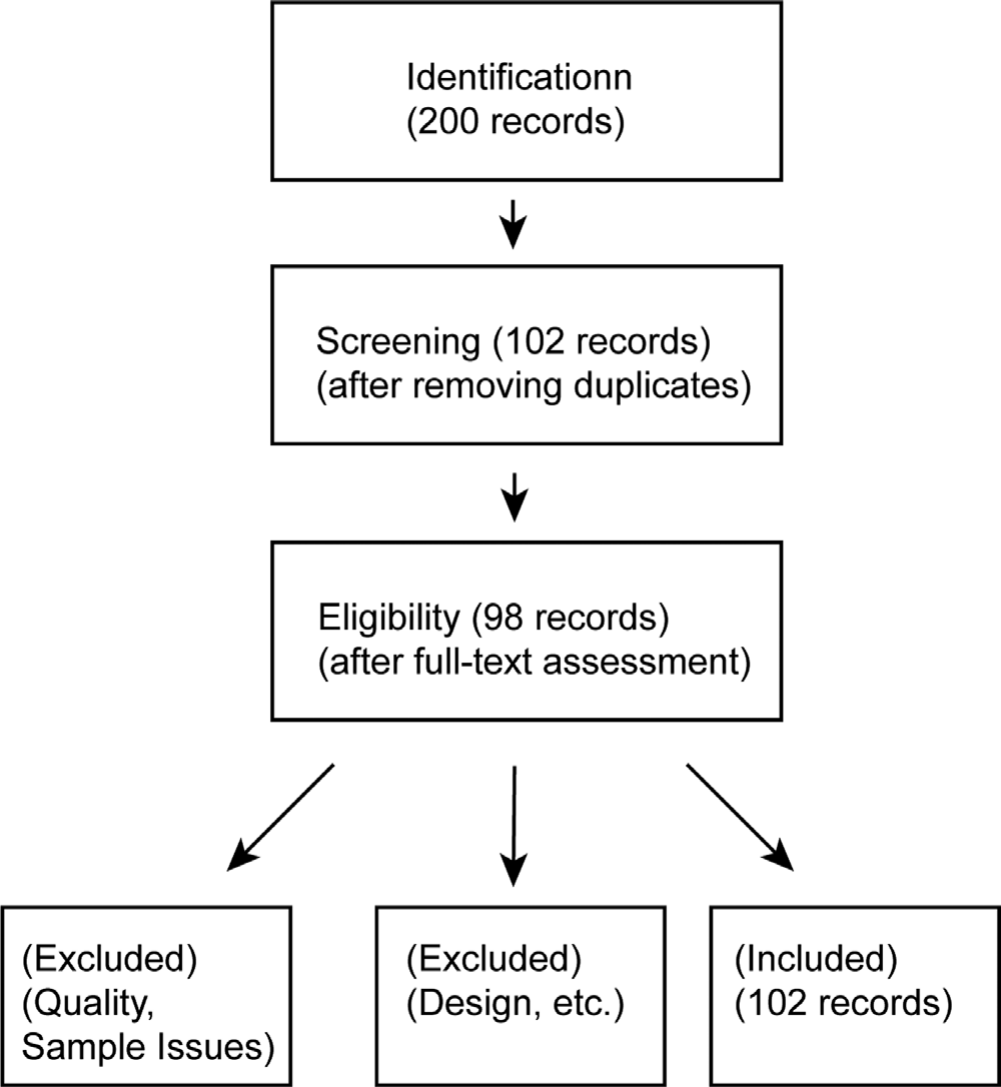
PRISMA figure include the search strategy, inclusion, or exclusion criteria.

A comprehensive literature search was conducted across multiple databases, including PubMed, Scopus, and the Web of Science, to identify relevant studies published in English between 2001 and 2024. The search terms included a combination of keywords such as “nanotechnology, Chinese medicine based-nanomaterials, coronary, cardiovascular diseases, and their synonyms”. The search was supplemented with manual searches of reference lists of key articles and relevant reviews to ensure comprehensive coverage. No language restrictions were applied, and only studies published in peer-reviewed journals were considered (Fig. 2).

### 2.2. Inclusion and exclusion criteria

Studies were included if they met the following criteria:

1. Published in peer-reviewed journals.
2. Reported on [insert relevant research topic or population].
3. Included original empirical data (quantitative or qualitative).
4. Published within the specified time frame (2001–2024).

Studies were excluded based on the following criteria:

1. Non-English language publications.
2. Reviews, meta-analyses, conference abstracts, and editorials.
3. Studies without original data or methodological issues (e.g., no control group or sample size too small).
4. Studies that did not meet the specified quality criteria after full-text assessment.

## 3. The application of conventional Chinese medicine nanoparticles in treating CHD

Based on their physical characteristics, nanoparticles can be classified as organic nanocarriers, such as liposomes, micelles, dendrimers, polymer nanoparticles, carbon nanotubes, and graphene, which are widely used for drug delivery because of their good biocompatibility and easy functionalization.^[33,34]^ Inorganic nanocarriers, such as silicon nanoparticles and gold nanoparticles, known for their excellent stability, high drug-loading capacity, and ease of surface modification, are utilized in drug delivery.^[35,36]^ They can be tailored to specific shapes, dimensions, and surface features to optimize their biodistribution and drug-release behavior.^[37,38]^ Many nanoparticles have been widely studied for drug delivery in CAD, and some common particle applications are described below (Table 1).

**Table 1:** Advantages and varieties of nanoparticles.

Type	Model/patient	Advantages
Liposomes	Atherosclerosis humans	Increase the half-life of circulation. Improved atherosclerotic macrophage targeting. No negative impact on markers related to metabolism
Polymers	Plaque rupture mouse	Spotted macrophages in the tissue of plaque. Control the inflammatory response of plaque. Decreased numbers of inflammatory cells. Successfully lower the quantity of fibrous caps. Maintain plaque tissue stabilization.
Polymers	Myocardial infarction rats	A smaller infarct. Boost the heart’s systolic function. Successfully prevent cardiac fibrosis. Enhanced targeting of infarct myocardium and cardioprotective properties.
Polymeric Micelles	Embolic rats	Spotted macrophages in the tissue of plaque. Control the inflammatory response of plaque. Decreased numbers of inflammatory cells. Successfully lower the quantity of fibrous caps. Maintain plaque tissue stabilization.

### 3.1. Liposomes

Liposomes, which are nanoscale vesicles consisting of natural or synthetic lipids, were one of the 1st nanocarriers used for drug delivery.^[39]^ They enhance the stability and bioavailability of drugs by mimicking the structure of biological membranes, while also reducing drug toxicity and side effects.^[40]^ Liposomes can encapsulate several drugs, including both hydrophilic and lipophilic drugs.^[41]^ They are characterized by low toxicity and the ability to evade immune cell recognition.^[42]^ They are efficient in sustaining the release of drugs and extending their presence in the body, thus enhancing their therapeutic effect with minimal side effects.^[43]^ The buildup of liposomal nanoparticles incorporating prednisolone in atherosclerotic macrophages was investigated in a rationalized, randomized, placebo-controlled clinical study.^[44]^ The results showed that these nano-particles greatly prolonged the circulating half-life and enhanced the targeting of atherosclerotic macrophages, with no adverse effects on cardiometabolic parameters.^[45]^ Liposomal nanoparticles encapsulating prostaglandin E1 are commonly used in treating myocardial infarction, angina pectoris, and other cardiovascular diseases.^[46]^ Prostaglandin E1 inhibits platelet aggregation, enhances vasodilation, increases microcirculation, dilates coronary arteries, and enhances myocardial perfusion.^[47]^ A randomized controlled study with 300 patients undergoing PCI for acute myocardial infarction demonstrated that the combination of prostaglandin E1 and *Salvia divinorum* diterpene IIa significantly improved cardiac performance and ventricular remodeling after PCI and reduced the occurrence of undesirable events.^[48]^ Baicalin, the main component of *Scutellaria baicalensis*, is widely used in the clinic for treating hypertension, infections, hepatitis, and other diseases, with common formulations, including tablets and injections.^[49]^ However, despite their wide application, *Scutellaria* preparations face issues such as poor oral absorption, low bioavailability, unstable aqueous solutions, and short half-life in the body.^[50]^ Xu et al utilized the reverse evaporation method to formulate baicalin into liposomes, and found that it could significantly prolong the retention period of baicalin in the body, with significant liver and lung targeting, allowing more drug accumulation at the lesion site, thereby enhancing therapeutic effects.^[51]^ Ginsenoside Rg1 has protective effects on the myocardium, regulates blood lipids, and is anti-atherosclerotic; however, it is easily degraded by intestinal bacteria and eliminated quickly in the blood.^[52]^ To enhance the targeting of ginsenoside in the body and improve its therapeutic effect, Zhou Hongwei studied the preparation of ginsenoside Rg1 liposomes.^[53]^ The findings indicated that ginsenoside Rg1 produced by the thin-film dispersion approach had an ideal encapsulation rate and bioavailability and could be fully formulated into drug-carrying liposomes.

### 3.2. Polymers

Polymer nanoparticles made from natural or synthetic polymers such as polyethylene glycol and polylactic-co-glycolic acid (PLGA) are widely employed in drug delivery because of their excellent biocompatibility, controllable degradability, and drug release characteristics.^[54]^ Upon degradation, they produce nontoxic oligomers as final products that are compatible with most drugs.^[55]^ Polymeric nanoparticles utilize their physical characteristics, such as size and large surface area to volume ratio, to enhance the cellular uptake and bioavailability of drugs.^[56]^ Drug release rates can be managed by adjusting the polymer composition, molecular weight, and cross-linking.^[57]^ Nevertheless, polymer nano-particles have drawbacks; for example, the natural polymer chitosan is incompatible with biofluids, resulting in degradation of the particles and decreased efficacy.^[58]^ However, the surface modification of nanoparticles can overcome this problem and enhance their biocompatibility and targeting ability. For example, in a mouse model of atherosclerosis, PLGA nanoparticles loaded with pioglitazone showed better therapeutic efficacy than free pioglitazone.^[59]^ According to Lin et al, using a nanoparticle-mediated delivery system, the fibrous cap is markedly reduced in number and thickness because modified PLGA nano-particles target monocytes or macrophages and activate receptors that promote macrophage differentiation, thereby modulating inflammation.^[60]^ Utilizing ion gel formulation, a rosuvastatin–chitosan nanocarrier system was developed and tested in vivo on a hypercholesterolemia rabbit model.^[61]^ Their recently released findings suggested that the nanoparticle group was more effective in lowering lipid levels than the rosuvastatin-only category.^[62]^ In addition, heart valve calcification was reduced in the nanoparticle group compared to that in the rosuvastatin alone group. PLGA nanoparticles carrying pitavastatin showed good safety and tolerability in clinical phase I/IIa trials in patients with chronic limb ischemia.^[63]^ This promising result indicates that PLGA nanoparticles are an effective strategy for the treatment of vascular diseases.

### 3.3. Gold nanoparticles

Gold nano-particles, as stabilized inorganic metallic nanocarriers, have numerous benefits suited for the release of cardioprotective drugs.^[64]^ Its main benefits are its low toxicity to other cells and its non-immunogenicity, which has led to its widespread use in the field.^[65]^ Because of their distinctive structural features, gold nanoparticles have a good ability to target ischemic tissues. Therefore, drug loading on these nanoparticles can effectively penetrate and build up in tissues, promoting rapid recovery of ischemic tissues and angiogenesis by delivering exogenous growth factors.^[66]^ To improve drug transport efficiency, metoprolol was complexed with gold nanoparticles that specifically targeted the beta-1 receptor. Compared to administration alone, this complex showed double the therapeutic effect in heart tissue subjected to heart failure, while adverse effects in other tissues were minimal.^[67]^ Polyethylene glycol-modified gold nanoparticles can reduce the size of infarcted myocardial tissue by mitigating necrosis and apoptosis in cardiomyocytes, study finds.^[68]^ Furthermore, they control inflammation via collagen deposition.^[69]^ Several studies have suggested the use of gold nano-particles or gold nano-particle polymers for the treatment of cardiovascular disorders. Although gold nanoparticles have demonstrated great promise as therapeutic agents against acute myocardial infarction in laboratory studies, additional research is needed to define their commercial use and application.

### 3.4. Polymeric micelles

Polymer micelles are self-assembled entities with a hydrophilic outer shell and a hydrophobic inner core, characterized by a special core-shell configuration, smaller dimensions, narrow particle size distribution, relatively stable structure, and high drug loading capacity.^[70]^ In addition, polymeric micelles improve the dissolution of difficult-to-solve drugs and facilitate their entry into target tissues. Li Ying et al used glycyrrhizic acid, a safe, nontoxic, and amphiphilic natural compound, as a polymeric material to prepare andrographolide-glycyrrhizic acid nanomicelles, which significantly enhanced the solubility and antitumor effect of andrographolide.^[71]^ Jin Jiahui and others have created nanopolymeric micelles with a blend of resveratrol oxide, polyethylene glycolated hydrogenated castor oil, and hyaluronic acid in the ratio of 0.1%:3.0%:0.2%, significantly improving its solubility in water. A transdermal absorption experiment using a Franz diffusion cell demonstrated good transdermal absorption performance, making it a candidate formulation for resveratrol aqueous solutions.^[72]^ However, polymeric micelles also have some drawbacks, such as poor stability of the micelle structure, and their physicochemical properties may change during lyophilization process.^[73]^ Therefore, it is necessary to add cryoprotectants to maintain physicochemical stability.

### 3.5. Advantages and prospects

#### 3.5.1. Enhancing bioavailability

Nano-delivery systems significantly enhance the bioavailability of drugs by improving their solubility and stability and increasing their concentration in target tissues or cells.^[74]^ This is particularly important for components of TCM that were originally difficult for the human body to absorb and utilize.

#### 3.5.2. Targeted delivery and controlled release

Nanocarriers can be designed with specific surface markers to precisely target disease-related cells or tissues.^[75]^ In addition, by controlling the rate and timing of drug release, the therapeutic effect of the drug can be maximized while potentially reducing damage to healthy tissues.

#### 3.5.3. Enhancing therapeutic effects and reducing side effects

By enhancing the specificity and bioavailability of drugs, nano-delivery systems can improve therapeutic efficacy without increasing dosage.^[76]^ Simultaneously, they reduce the accumulation of drugs in non-target tissues, thereby alleviating the side effects.

## 4. Preparation and characteristics of TCM nanoparticles

According to incomplete statistics, there are currently 34 types of TCM formulations, with more than 20 commonly used types.^[77]^ Although classical formulations, such as tablets, injections, ointments, pills, capsules, suppositories, and plasters still occupy the majority of the market share, the integration of new drug delivery systems and modern technologies with traditional formulations has injected new vitality into traditional prescriptions.^[78]^ Recently, with the progression of several modern formulation methods, a plethora of TCM products represented by liposomes and microcapsules has emerged, making significant progress in improving the palatability of TCM, enhancing stability, achieving sustained, controlled release, and targeted therapy, among other special purposes.^[79]^ Microencapsulated TCM can mask the unpleasant smell and taste of drugs, improve stability, prevent drug inactivation in the stomach or reduce gastric irritation, minimize the variability in combination with compound medicines, achieve sustained or controlled release purposes, and enhance drug targeting, especially for arterial thrombosis therapy, etc.^[80]^ Improving the level of Chinese medicine formulation is undoubtedly a shortcut to modernizing Chinese medicine formulation.^[81]^ The investigation and application of novel technologies in drug formulations for cardiovascular TCM formulations and the development of new formulations can significantly improve the slow onset of action, inconvenience, unstable efficacy, and difficulty in quality control of traditional TCM formulations.^[82]^ The different TCM formulations can be categorized as follows (Table 2).

**Table 2:** Traditional Chinese medicine formulations.

Type	Model/patient	Advantages
Aromatic patch	Plaque rupture mouse	Avoids gastrointestinal degradation and first-pass effect on liver, and has the advantages of fast onset, low dose and little side effect.
Microcapsules and microspheres	Plaque rupture mouse	Slow-release effect, targeting effect, larger drug loading and biological activity.
Microemulsion	plaque rupture mouse	Good thermodynamic stability, good dispersion, easy absorption, soluble lipophilic drugs, prevent drug hydrolysis.
Clathrate compound	Plaque rupture mouse	Increase the stability of the drug, mask unpleasant odors, reduce irritability, and improve the solubility of insoluble drugs.

### 4.1. Aromatic patch

Nasal inhalation aroma patches, also known as nasal aroma patches, are a new formulation of inhalation preparations.^[83]^ They utilized a patch as the adherence model, with volatile aromatic drugs applied below the nose at the “Ren Zhong” area, where the drug in gas form is actively inhaled through the nose into the lungs to exert local or systemic therapeutic effects.^[84]^ Unlike traditional patches, these are not transdermal formulations but use the adhesive layer of the patch for attachment purposes only.^[85]^ Nasal inhalation aroma patches avoid gastrointestinal degradation and 1st-pass impact on the liver, offering the advantages of rapid onset, low dosage, and minimal side effects.^[86]^ Furthermore, the use of nasal inhalation aroma patches at night ensures patient safety during nighttime hours, providing a timed release of medication. Su Bing nasal patches have a good anti-hypoxic effect, good in vivo and in vitro correlation, and safety, with rapid onset, long duration of action, and definitive therapeutic effects in treating cardiovascular diseases, showing great potential for development.^[87]^ Coronary Suhe pills are effective in treating angina pectoris and chest tightness, but their aristolochic acid content is nephrotoxic.^[88]^ Xiaodong et al formulated it into Coronary Suhe Nasal Inhalation Aroma Patches, where the drug is inhaled through the nose, quickly reaching the lungs and heart, providing better therapeutic effects for angina pectoris and chest tightness while avoiding the nephrotoxicity of aristolochic acid.^[89]^

### 4.2. Microcapsules and microspheres

Microspheres are tiny spherical entities created by the dissolution or dispersion of a drug in a polymer matrix, with common particle sizes ranging from 1 to 40 μm, which are matrix-type skeletal particles.^[90]^ Microcapsules utilize natural or synthetic polymeric compound materials as capsule materials, encapsulating solid or liquid drugs to form tiny reservoir-type particles, generally ranging from a few micrometers to 400 μm in size.^[91]^ Drugs encapsulated in microcapsules not only have a slow-release effect but also a targeting effect. Compared with microspheres, microcapsules have a larger drug load and biological activity. Danshen is effective in reinforcing the heart, expanding coronary blood vessels, and preventing thrombosis and is commonly used in the treatment of cardiovascular diseases.^[92]^ Experiments illustrated that tanshinone could enhance the expression of endothelial nitric oxide synthase (eNOS) protein and improve the level of eNOS phosphorylation, dilating blood vessels. The mechanism of tanshinone in dilating blood vessels and lowering blood pressure may be related to the regulation of eNOS.^[93]^ To enhance the efficacy of Danshen and protect its active components from digestion in the gastrointestinal tract or decomposition by bacteria, Xiao et al created Danshen microcapsules using high-speed centrifugal shearing ultrafine grinding technology combined with spray drying technology, significantly improving its bioavailability in the body.^[94]^ Ligustrazine is a new drug for treating thrombosis and other diseases and is capable of significantly increasing coronary flow, lowering arterial pressure, and reducing coronary resistance.^[95]^ Lin Yaling and others, using star-shaped poly-L-lactic acid (sPLLA) al. prepared ligustrazine phosphate (LP) sPLLA drug-loaded microspheres (sPLLA/LP) using an emulsification-solvent evaporation method. LP combines well with sPLLA, and after 7 days of sustained release, sPLLA begins to partially degrade, demonstrating a good sustained-release effect.^[96]^

### 4.3. Microemulsion

A microemulsion is a water-in-oil (O/W) or oil-in-water (W/O) type dispersion, with a particle size range of 10 to 100 nm, formed from combinations of 2 immiscible fluids in the presence of a surfactant and co-surfactant.^[97]^ Microemulsions are novel drug carriers that can be used for immediate release, sustained release, and targeted drug delivery strategies to improve drug bioavailability. Microemulsions can be utilized as drug carriers in oral liquid formulations and transdermal drug delivery systems, offering the advantages of thermodynamic stability, good dispersibility, facilitation of absorption, solubilization of lipophilic drugs, and prevention of drug hydrolysis.^[98]^ *Astragalus*, a common TCM, is often used clinically to treat hypertension with lower-limb edema in the elderly and ischemic heart disease.^[99]^ Yu Dongsheng prepared a concentrated microemulsion of astragalus injection solution using the pseudo-ternary phase diagram method, resulting in a stable drug formulation with a sustained-release effect.^[100]^ Puerarin is the major active ingredient of *Pueraria mirifica* and is used clinically for the treatment of CHD, angina pectoris, hypertension, and diabetes mellitus in commercial formulations including tablets, capsules and injections.^[101]^ The low water solubility of puerarin leads to its low bioavailability, poor efficacy, slow onset, and potential for allergic reactions and hemolysis. Changfeng studied the bioavailability of oral puerarin microemulsion in rats and found that compared to puerarin solution, the microemulsion promoted the absorption of puerarin, significantly enhancing its bioavailability.^[102]^

### 4.4. Clathrate compound

Inclusion compounds are molecular complexes in which a molecule is encapsulated in the cavity configuration of a different molecule; β-cyclodextrin inclusion compounds are the most common inclusion compounds.^[103]^ Encapsulation can increase drug stability, mask unpleasant odors, reduce irritability, and improve the solubility of insoluble drugs.^[104]^ Paeonol, found in commonly used Chinese medicines, such as peony bark and peony, has significant antihypertensive and analgesic effects.^[3]^ However, the water solubility of phenol is low, making it difficult to disperse and absorb it after ingestion. After encapsulation with β-CD, the water solubility of the inclusion compound increased, the drug release rate accelerated, and the bioavailability was enhanced. Dong quai has excellent antihypertensive effects, with volatile oils being the main pharmacologically active component.^[105]^ Xiangliang et al encapsulated Dong quai volatile oil with β-cyclodextrin, and experimental results showed that the composition of the volatile oil did not change before and after encapsulation, and gas chromatography proved that the inclusion compound has good stability.^[106]^

## 5. Future development directions

Despite significant progress in the application of nanomedicine, particularly TCM nanoparticles, for the treatment of CHD, several critical research gaps remain in the field. First, while studies have demonstrated the potential of TCM nanoparticles to improve cardiovascular function, reduce inflammation, and promote vascular regeneration in CHD patients, most of the research is still in the preclinical stage, and large-scale, long-term clinical trial data are lacking. Thus, the effective translation of laboratory research into clinical treatment remains a key challenge.

Second, the efficacy and safety of nanoparticles for CHD treatment have not been fully validated. Existing clinical studies often focus on individual drugs or therapeutic strategies, and a comprehensive evaluation of nanoparticle drug delivery systems is still limited. In particular, issues related to the toxicology, safety, and side effects of long-term nanoparticle use remain inadequately addressed, and more research data are needed. Furthermore, overcoming challenges related to the biodistribution, elimination, and targeted delivery of nanoparticles in the body continues to be a major bottleneck in nanomedicine research.

Another key gap is the standardization and preparation methods of TCM nanoparticles. Although some progress has been made in the development of preparation techniques, issues such as process controllability, batch-to-batch consistency, and scalability remain, which seriously hinder the widespread clinical application of these nanoparticles. Future research should focus on developing more efficient, environmentally friendly, and scalable production techniques for TCM nanoparticles to ensure consistent quality and efficacy.

## 6. Future directions

1. Clinical trials and efficacy evaluation: more high-quality, large-scale, multicenter clinical trials are needed to verify the efficacy and safety of TCM nanoparticles in CHD treatment. Special attention should be paid to the side effects and biocompatibility of nanoparticles in long-term therapy.
2. Targeting and drug delivery systems: improving the targeting ability and drug delivery efficiency of nanoparticles is crucial to ensure more precise action at the site of disease and to minimize side effects on healthy tissues. Additionally, the development of multifunctional nanoparticle carriers that integrate various therapeutic approaches (e.g., drug therapy and gene therapy) for synergistic effects should be explored.
3. Standardization and scalable production: research should focus on developing efficient, standardized production techniques for TCM nanoparticles, ensuring batch-to-batch consistency, and the production of high-quality drug products that meet the demands of clinical use.
4. Personalized medicine and precision therapy: with advancement of personalized medicine, future research could combine nanomedicine with genomics, proteomics, and other technologies to develop tailored treatment plans for specific patient populations. By targeting treatments more precisely, therapeutic efficacy can be improved, while minimizing adverse effects.
5. Multimodal therapy and combination approaches: future studies should explore combining TCM nanoparticles with other treatment modalities (such as traditional drugs and stem cell therapy) to create multimodal therapeutic strategies. This approach could enhance overall therapeutic effects and provide more comprehensive solutions for CHD treatment.

In conclusion, although nanomedicine, particularly TCM nanoparticles, holds great potential for the treatment of CHD, several challenges remain. Future research should strengthen the integration of basic and clinical studies, address technical bottlenecks in nanoparticle applications, and promote their translation into clinical practice.

## 7. Discussion

This review provides a comprehensive and up-to-date evaluation of the role of nanomedicine, particularly TCM nanoparticles, in the treatment of CHD.^[102]^ While several reviews have addressed the broader application of nanomedicine in cardiovascular diseases, few have specifically focused on the integration of TCM nanoparticles in CHD treatment, which sets this review apart.

Many existing reviews in this field have predominantly concentrated on the general properties of nanomedicines, their biocompatibility, and their pharmacokinetics in treating CHD. However, these reviews often overlook or only briefly mention the specific challenges and applications of TCM-based nanoparticles. In contrast, this review highlights the preparation methods, surface properties, drug release kinetics, and mechanisms of action of TCM nanoparticles in detail, providing a more nuanced understanding of their potential therapeutic advantages.

Additionally, while many reviews emphasize the clinical potential of nanomedicine in CHD treatment, there is often insufficient discussion on the challenges faced by nanomedicine in clinical settings, such as issues related to large-scale production, standardization, and safety concerns. This review addresses these challenges and explores potential solutions, emphasizing the future prospects of nanomedicine in personalized and multimodal therapies.

Moreover, the scope of this review is broader, offering insights into the epidemiological aspects of CHD, which is crucial for understanding the full impact of nanomedicine in this field. By combining both traditional therapeutic strategies and emerging nanomedicine-based approaches, this review provides a holistic view of the current state and future directions of CHD treatment.

In summary, compared with existing reviews, this review offers a more in-depth exploration of the specific role of TCM nanoparticles in treating CHD, along with a thorough discussion of the clinical challenges and future opportunities for nanomedicine in cardiovascular care. This makes a valuable contribution to the literature and provides a unique perspective on the integration of nanomedicine and TCM in modern cardiovascular therapy.

## 8. Conclusions

In this review, we provide an in-depth discussion of the progress of nanomedicine in treating CHD and draw the following conclusions: research on nanomedicine for the treatment of CHD has yielded numerous positive results. Several studies have shown that the use of nanotechnology carriers to improve the bioavailability and targeting of active components in TCM can significantly improve cardiovascular function in patients with CHD and decrease the incidence of cardiovascular incidents. Nanoscale TCM particles exhibit good biocompatibility and safety in treating CHD, effectively reducing the risk of thrombosis, protecting the myocardium from ischemia–reperfusion injury, and possessing anti-inflammatory, antioxidant, and lipid-regulating effects. The main advantages of TCM nanoparticles in the treatment of CHD are targeting and efficiency; nanoscale TCM particles can precisely release drugs through targeted delivery systems, increase drug concentration at the lesion site, and enhance efficacy. Achieving controlled release: nanoparticles can achieve sustained drug release, prolong the duration of drug efficacy, and improve therapeutic stability. Simultaneously, they alleviate side effects; nanocarriers can reduce drug dosage and frequency, thereby reducing the occurrence of adverse reactions and side effects. However, our study has the following limitations: small sample size and short study duration: current research is mainly focused on laboratory experiments and small-scale clinical trials, with a lack of long-term, large-sample, and randomized controlled trials. Lack of harmonized standards and norms: missing harmonized standards and norms in formulation, quality control, dosage, etc of nanomedicine for the treatment of CHD, which hinders its promotion and popularization in clinical applications. Need for in-depth study of the mechanisms of action: although studies have shown that nanoscale TCM particles have multiple effects in treating CHD, their detailed mechanisms of action still require further research. Future research directions include establishing a more complete research system and standardized formulation processes, conducting more clinical trials, and exploring the mechanisms of action of nanomedicine to further improve its efficacy and safety for treating CHD.

## Author contributions

**Investigation:** Yuhang Cheng, Miao Yu, Chenwei Dang.

**Project administration:** Huijun Chen.

**Resources:** Huijun Chen.

**Supervision:** Huijun Chen.

**Writing – original draft:** Yuhang Cheng, Miao Yu.

**Writing – review & editing:** Yuhang Cheng, Huijun Chen.
